# Targeted DNA vaccines eliciting crossreactive anti-idiotypic antibody responses against human B cell malignancies in mice

**DOI:** 10.1186/1479-5876-12-207

**Published:** 2014-07-25

**Authors:** Pier Adelchi Ruffini, Audun Os, Riccardo Dolcetti, Geir E Tjønnfjord, Ludvig A Munthe, Bjarne Bogen

**Affiliations:** 1 Department of Immunology, Centre for Immune Regulation, University of Oslo, Oslo University Hospital, Rikshospitalet, NO-0424 Oslo, Norway; 2 Department of Haematology, Oslo University Hospital, Rikshospitalet, NO-0424 Oslo, Norway; 3 Cancer Bio-Immunotherapy Unit, Centro di Riferimento Oncologico, Aviano, PN, Italy; 4 Institute of Clinical Medicine, University of Oslo, Oslo, Norway; 5 K.G. Jebsen Centre for Research on Influenza Vaccines, University of Oslo and Oslo University Hospital, Oslo, Norway

**Keywords:** Lymphoma, Cancer vaccine, Idiotype, Chemokine, CLL

## Abstract

**Background:**

Therapeutic idiotypic (Id) vaccination is an experimental treatment for selected B cell malignancies. A broader use of Id-based vaccination, however, is hampered by the complexity and costs due to the individualized production of protein vaccines. These limitations may be overcome by targeted DNA vaccines encoding stereotyped immunoglobulin V regions of B cell malignancies. We have here investigated whether such vaccines might elicit cross-reactive immune responses thus offering the possibility to immunize subsets of patients with the same vaccine.

**Methods:**

Fusion vaccines targeting patient Id to mouse Major Histocompatibility Complex (MHC) class II molecules (chimeric mouse/human) or chemokine receptors (fully human) on antigen-presenting cells (APC) were genetically constructed for two Chronic Lymphocytic Leukemia (CLL) patients and one prototypic stereotyped B-cell receptor (BCR) commonly expressed by Hepatitis C Virus (HCV)-associated Non Hodgkin Lymphoma (NHL). The A20 murine B lymphoma cells were engineered to express prototypic HCV-associated B cell lymphoma BCR. Anti-Id antibody responses were studied against stereotyped and non-stereotyped BCRs on CLL patients’ cells as well as transfected A20 cells.

**Results:**

DNA vaccination of mice with Id vaccines that target APC elicited increased amounts of antibodies specific for the patient’s Id as compared with non targeted control vaccines. Anti–Id antibodies cross-reacted between CLL cells with closely related BCR. A20 cells engineered to express patients’ V regions were not tumorigenic in mice, preventing tumor challenge experiments.

**Conclusions:**

These findings provide experimental support for use of APC-targeted fusion Id DNA vaccines for the treatment of B cell lymphoma and CLL that express stereotyped BCRs.

## Background

B cell malignancies express a highly tumor-specific antigen, the variable (V) regions of the monoclonal immunoglobulin (Ig), which contain antigenic determinants called idiotopes collectively known as idiotype (Id). Protein Id vaccination has been pursued as a therapeutic approach to B cell malignancies over the last 20 years [1]. Immunologic and clinical responses have been detected [1], whereas demonstration of clinical benefit is so far limited to follicular lymphoma [2,3].

Because of the very nature of the antigen (Ag) (i.e., Id), large scale clinical application of protein Id vaccination is limited by the need to prepare a custom-made vaccine for each and every patient. Overcoming this problem, DNA vaccination holds promise to streamline tailor-made vaccine manufacture by circumventing the need for purification of Ig protein (or derivatives thereof), conjugation to carrier protein (e.g. KLH) and administration of adjuvants. However, although effective in rodents, DNA vaccination has met with limited success thus far in humans due to low potency of vaccines [4]. The poor immunogenicity of DNA vaccination can be improved by several means such as improved vector design and efficient electroporation [5]. Another strategy is based on the finding that targeting of Ag to antigen-presenting cells (APC) enhances immunogenicity, as shown for chemical antibody (Ab)-Ag conjugates [6,7] and Ab-Ag fusion proteins [8,9]. Thus, we [10] and others [11,12] cloned DNA constructs encoding proteins that target Ag to APC. When such constructs were injected *s.c.* or *i.m.*, combined with electroporation, transfected host cells secreted fusion proteins that targeted APC for enhanced immune responses [10]. In our previous studies, we used homodimeric Ig-based vaccines (Vaccibodies, VB), each chain consisting of a targeting unit, a dimerization unit and an antigenic unit. The dimerization unit consists of a shortened hinge region from hIgG3 whereas the N-terminal targeting unit can consist of either single chain fragment variable (scFv) specific for surface molecules on APC such as mouse MHC class II [10], mouse CD40 [13], human TLR2 and CD14 [14], or natural ligands such as the mouse chemokines CCL3 (mCCL3) and CCL5 (mCCL5) [15] and human CCL3 [16]. Depending on targeting strategy, such VB proteins had a 10–10,000 fold increased efficiency to stimulate CD4^+^ T cells *in vitro* in mice [10,13,15,16] and humans [14,16]. Moreover, DNA Vaccibodies elicited superior antibody and T cell responses in mice, as well as greatly enhanced tumor protection [10,13,15,16]. In a stepwise, translational endeavour, the first fully murine Vaccibodies [10] have been extended to chimeric murine/human Vaccibodies, including tailor-made Vaccibodies for multiple myeloma patients [17].

A complementary strategy to streamline clinical Id vaccination is to exploit the high similarity of Ig V regions expressed by molecularly identified subgroups of patients with B cell malignancies. For example, the molecular characterization of Hepatitis C Virus (HCV) related lymphomas showed that more than 70% of these cases expressed either IGKV3-20 or IGKV3-15 light chains [18-20], with a high degree of homology between individual lymphomas. Moreover, IGHV1-69 is expressed as the partner of IGKV3-20 or IGKV3-15 in up to 70% of HCV-related lymphomas [18,20]. Such commonly expressed B cell receptors (BCR) are called stereotyped receptors. Stereotyped BCRs are found also in several non HCV-associated B cell malignancies, such as MALT lymphomas [21-23] and Chronic Lymphocytic Leukemia (CLL) [24-26]. The analysis of VH CDR3 in more than 7000 VH (IGHV-IGHD-IGHJ) sequences from patients with CLL has established that CLL comprises two distinct categories: one with stereotyped and the other with heterogeneous BCR, in an approximate ratio of 1:2 [27]. Thus, it could be envisioned that a number of off-the-shelf Id vaccines for molecularly identifiable subgroups of patients could be developed, obviating the need to tailor-make Id-vaccines for every patient. Although it is not known whether these Ids are immunogenic in the majority of patients, such off-the-shelf Id vaccines could cover up to 30% of patients with selected B cell malignancies, thus affording substantial savings in time and costs associated with Id vaccine manufacture.

On these premises, we have here produced fully human chemokine-Id fusion DNA Vaccibodies which due to cross-species reactivity of chemokines could be tested as DNA vaccines in mice. Moreover, using a panel of CLL patients’ cells and a mouse model for HCV-associated B cell lymphomas we explored the possibility of inducing cross-reactive anti-Id antibody responses following immunization with VB expressing a stereotyped B cell receptor.

## Methods

### Patient material

Patients diagnosed with CLL (see Table 1) were seen at the Department of Haematology outpatient clinic, Oslo University Hospital, Rikshospitalet, Oslo, Norway. Blood samples from 5 patients were procured following written informed consent using protocols approved by the Regional Committee for Medical and Research Ethics, South-East Norway. Blood samples were procured in tubes containing ACD as anticoagulant. Experiments were conducted on purified mononuclear blood cells.

**Table 1:** Characteristics of CLL patients’ BCR

**CLL pt.**	**isotype**	**IGHV**	**IGHD**	**IGHJ**	**IGLV**	**IGLJ**
103	IgMλ	3-48*02	2-2*01	4*01	3-21*01	3*02
106	IgMλ	3-23*01	4-23*01	4*02	3-21*01	1*01
107	IgMκ	3-30*02	4-17*01	5*02	4-1*01	4*01
111	IgMλ - κ	3-53*01	n.a.	1*01	3-21*01 λ	3*02
					3-20*01 κ	1*01
116	IgMκ	4-59*01	2-15*01	2*01	3-20*01	1*01

n.a.: no results in IMGT/V-QUEST.

### Flow cytometry

Cells were stained with primary reagents and appropriate secondary reagents or control as indicated. The following biotinylated mAbs were used: anti human IgG (HP6017, Zymed), anti mouse IgD (TIB149, ATCC), anti mouse Ck (clone 187.1), anti mouse IgG1^a^ (clone 10.9, BD Pharmingen), anti mouse IgG2a^a^ (clone 8.3, BD Pharmingen), anti mouse IgG2a^b^ (clone 5.7, BD Pharmingen). Quantification of surface antigen on CLL cells was performed using mouse mAbs targeting human λ (clone 4C2) and human κ L chains (clone A8B5), and human IgM (clone 1030) from Diatec, Oslo, Norway, and the bead based Cellquant Calibrator kit (BioCytex, Marseille, France) according to the manufacturer’s guidelines [28]. Cells (20,000) were acquired on a FacsCalibur (BD). Flow cytometry files were analyzed on CellQuest (BD) and Weasel v3.0 (http://www.wehi.edu.au).

### Mice and cell lines

BALB/c mice were obtained from Taconic (Ry, Denmark). B10.D2.C-TCRα^a^/Bo (H-2^d^, Ig haplotype IgH-C^b^) mice were bred in house. These congenic mice are identical to B10.D2 except being congenic for the TCRα^a^ region [29]. The studies were approved by the National Committee for Animal Experiments (Oslo, Norway). HEK 293E cells were from ATCC. The murine lymphoma Esb/MP cells [30] were kindly provided by Jo Van Damme (Leuven, Belgium). The human Burkitt’s lymphomas DG-75 [31] and PA682 [32] were obtained from ATCC and kindly provided by Keith Thompson (Oslo, Norway), respectively.

### Identification of V_**H**_ and V_**L**_ genes from CLL patients and assembly of scFv

V_H_ and V_L_ tumor-specific genes were cloned from preparations of cDNA from PBMCs of each patient. Tumor-specific transcripts were identified by PCR as previously described [33].

Sequence data were analyzed using the IMGT database (http://www.imgt.org) and the IMGT/V-QUEST tool [34]. Nucleotide sequences were aligned using BLAST (http://blast.ncbi.nlm.nih.gov/). Amino acid sequences were aligned using clustal omega (http://www.ebi.ac.uk/Tools/msa/clustalo/) and GeneDoc (http://nrbsc.org/gfx/genedoc/) for analysis of conservative, semi-conservative and non conservative changes. Tumor-specific V_H_ and V_L_ genes from CLL patients were arranged in a single chain variable region fragment (scFv) in VH-VL orientation by PCR Soeing (Synthesis by Overlap Extension) essentially as described previously [33].

### Assembly of stereotyped scFv for Hepatitis C virus-associated B cell lymphomas

Plasmids encoding V (D) J_H_ (VH1-69, VD3-22, VJ4) and VJ_κ_ (VK3-20, VJ1) sequences from patients 1 and 2, respectively, were kindly provided by V. De Re (Aviano, Italy) and assembled into scFv as described above. This association has been shown to be representative of stereotyped Id molecules expressed by up to 70% of B cell malignancies associated with HCV chronic infection [18,20].

### Assembly of patient-specific and cross-reactive Vaccibodies

The resulting scFv from individual CLL patients or the prototypic stereotyped BCR from HCV-associated B lymphoma were cloned C-terminal as antigenic units into the previously described VB format [10]. In Vaccibodies used in this study, the N-terminal targeting unit consisted either of a mouse scFv from the 14-4-4S mAb specific for I-E mouse MHC class II molecules [10], or the human chemokine LD78β (CCL3-L1) which binds CCR1, CCR3 and CCR5 [16]. Control non-targeted Vaccibodies encoded a mouse scFv specific for the hapten NIP (5-iodo-4-hydroxy-3-nitrophenylacetyl), an antigen which is not found in the mouse tissue [10]. The dimerization unit consisted of a shortened hinge (h1 + h4) and C_H_3 of human IgG3 [10].

### Vaccine protein production and assessment of targeting properties

Expression and function of chimeric VB constructs was determined on supernatants from transiently transfected HEK 293E cells. To comparatively measure concentration of Vaccibodies expressing different targeting and antigenic units, an ELISA detecting the presence of human IgG3C_H_3 in the dimerization unit was set up: mAb MCA878G (binds human IgG3C_H_3, AB Serotec) as coat and biotinylated mAb HP6017 (binds to a different epitope in human IgG3C_H_3) for detection. Binding to MHC class II was verified by admixing I-E^d^-specific VB-containing supernatants and BALB/c (I-E^d+^) A20 B lymphoma cells. Bound VB proteins were detected as previously described [17]. NIP-specific Vaccibodies were tested for their ability to bind to NIP-BSA (conjugated in-house) as previously described [17]. Chemotactic activity of LD78β (CCL3-L1) Vaccibodies on the mouse Esb-MP T cell lymphoma was tested by a transwell plate (Corning), as previously described [16]. The results (mean + SE of duplicate samples) are presented as chemotactic index, defined as the fold increase in the number of migrating cells in the presence of chemotactic factors over the spontaneous cell migration (i.e., in the presence of medium alone).

### Purification of patient tumor Ig protein

Heterohybridomas secreting tumor-specific Ig were generated from PBMCs of two patients with CLL by standard procedures [35]. Hybridomas were screened by ELISA for expression of an Ig of expected H and L-chain isotypes. H-chain of hybridomas was verified by V_H_ sequencing. Hybridomas with V_H_ sequence fully matching that retrieved from patients’ CLL cells were selected for further study.

### Generation of mouse B lymphoma cells (A20) that express a stereotyped BCR of HCV-associated B cell lymphomas

V (D) J_H_ regions from patient 1 (V_H_P1) and VJ_L_ regions from patient 2 (V_κ_P2) were cloned into independent vectors that had been developed for membrane expression (but not secretion) of mouse IgDκ [36]. In particular, V_H_P1mIgDpLNOK vector expressing G418 resistance contains downstream of V_H_P1 the murine germline IgD sequence (IgD^a^ allotype) in which the 3’ secretory exons had been eliminated, whereas V_κ_P2 was cloned in the pMUSmCκ expressing mouse constant κ region and zeocin resistance (Tuva Hereng and Bjarne Bogen, unpublished) to generate V_κ_P2MUSKAP.

A20 BALB/c B lymphoma cells that express an endogenous IgG2aκ and MHC class II (including I-E^d^) were transfected with either or both vectors by electroporation, grown in selection medium (G418 and/or zeocin), and cloned by limiting dilution. To screen for A20 transfectants expressing patient V_H_, cells were stained with anti-IgD TIB149 (ATCC). Transfectants were selected by flow sorting for high IgD expression followed by cell culture and re-sorting. Expression of VH1-69 and VK3-20 was assessed by RT-PCR using the following primers: for VH1-69, forward GTGCAGCTGGTGCAGTCT and reverse TCCCTGGCCCCAATAGAAGT; for VK3-20 forward TTGTGTTGACGCAGTCTCCAG and reverse TTGATTTCCACCTTGGTCCCT.

As a negative control, we used an A20 cell that expressed IgDκ with V regions derived from the unrelated syngeneic Ab2-1.4 hybridoma [36].

### Mouse immunization

VB plasmids were purified with Endofree® Plasmid Mega Kit (Qiagen). 25 μL solution of 0.5 mg/mL VB DNA in sterile 0.9% NaCl (total 25 μg per mouse) was injected intradermally in the lower back of mice, on both sides, followed by electroporation using Derma Vax™ (Cyto Pulse Sciences, MD, USA). Groups consisted of 3 to 7 mice.

### Measurement of antibody responses and assessment of specificity of anti-scFv antibodies

Blood samples were obtained at different time-points from the leg vein of vaccinated mice. Sera were tested by ELISA for reactivity against the patients’ CLL-derived monoclonal IgM or control isotype-matched IgM, or human IgG3, as coat. Bound antibodies were detected by either biotinylated mAb 187.1 (detects murine Cκ), anti-mouse IgG1^a^ (clone 10.9 BD Pharmingen) or anti-mouse IgG2a^a^ (clone 8.3 BD Pharmingen). The endpoint titres were recorded as the final serum dilution giving a signal above a fixed concentration of alkaline phosphatase-conjugated goat anti-human IgM (Sigma) or biotinylated mouse anti-human IgG (HP6017).

Sera were also used to stain PBMC from different CLL patients. Bound mouse antibodies were detected with biotinylated mAb 187.1 followed by streptavidin PerCP. Surface Ig expression by CLL cells was assessed by CellQuant (Biocytex), measuring the average Ig surface number by analyzing 20,000 cells.

Sera from immunized BALB/c or B10.D2.C-TCRa^a^/Bo (H-2^d^ IgH-C^b^) mice were used to stain A20 transfectants expressing human IGHV1-69, or human IGKV3-20, or both. Following blocking with PBS with BSA and heat-inactivated rat serum, transfectants were admixed with sera. Bound mouse antibodies were detected with biotinylated anti-mouse IgG1^a^ for BALB/c sera or anti-mouse IgG2a^b^ for B10.D2.C-TCRa^a^ sera.

### Injection of transfected A20 cells in immunocompetent mice

BALB/c mice were injected *s.c.* with 3 × 10^6^ parental A20 cells or with A20 cells stably transfected with either VH1-69, or VK3-20 or both, and followed up for tumor growth.

### Statistical analysis

Analysis of variance and regression analysis were conducted on all treatment arms. Results are presented as comparison of slopes of the antibody response in different treatment arms across serum dilutions.

## Results

### Identification and assembly of CLL-derived V genes into Vaccibodies

**Figure 1:**
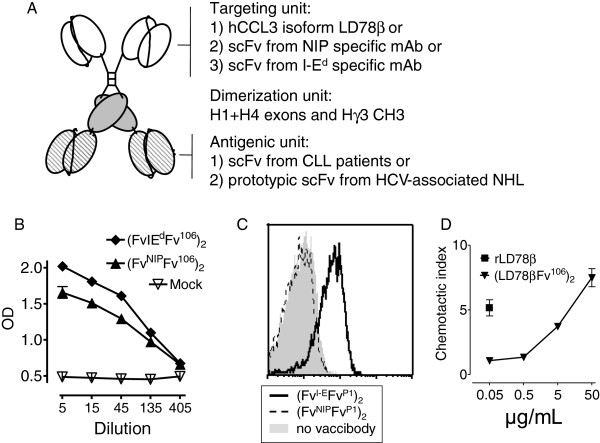
**Characterization of fusion vaccines used in this study. (A)** Schematic structure of a homodimeric vaccine fusion protein (Vaccibody, VB). Targeting, dimerization and antigenic units are indicated, as are the different moieties expressed in the various units. MHC class II targeted (I-E^d^-specific) and non-targeted (NIP-specific) constructs are denoted (Fv^I-E^Fv^Pn^)_2_ and (Fv^NIP^Fv^Pn^)_2_, respectively, whereas LD78β VB are denoted (LD78βFv^Pn^)_2._ where Pn indicates patient number. **(B)** Detection of VB proteins in supernatants of transfected HEK 293E cells in an ELISA specific for hγC_H_3. **(C)** Binding of MHC class II-targeted VB to I-E^d+^ A20 cells. **(D)** LD78β VB displays dose-dependent chemotactic activity on lymphocytic Esb/MP cells. Recombinant LD78β was used as positive control.

The characteristics of the patients’ CLL cell BCR included in this study are presented in Table 1. The CLL-specific V regions were identified in each case as identical VDJ_H_ and VJ_L_ sequences repeatedly obtained after cloning of PCR products. The corresponding scFv was then assembled for two patients (CLL106 and CLL107) and cloned into the antigenic unit of various VB scaffolds. According to previously published results on other Vaccibodies [10,13-17], transiently transfected HEK 293 cells were expected to secrete homodimeric fusion proteins consisting of *i*) two targeting units: either mouse scFv specific for mouse MHC class II molecules (I-E^d^), or the hapten NIP (non targeted negative control), or two human CCL3 (LD78β isoform) chemokine moieties, *ii*) two dimerization units: human hinge-CH3 held together by disulfide bonds and non-covalent interactions, and *iii*) two antigenic units: either human scFv of CLL origin (patients CLL106 and CLL107) or prototypic stereotyped V_H_ and V_L_ associated with HCV-associated B lymphomas (Figure 1A). ELISA on supernatants of transiently transfected 293E cells demonstrated similar levels of secretion of the fusion protein for each construct (Figure 1B and data not shown). The targeting units of the respective bivalent VB proteins retained their functional properties since anti-MHC II VB bound MHC class II + ve A20 cells (Figure 1C) and LD78β VB chemoattracted mouse Esb/MP cells (Figure 1D). Non targeted anti-NIP VB neither bound A20 cells (Figure 1C) nor chemoattracted Esb/MP cells, whereas it bound NIP-BSA in ELISA (data not shown). Taken together, these results of Figure 1 are consistent with previous extensive characterizations of VB molecules expressing other antigenic units [10,13-17].

### Analysis of the antibody responses induced by DNA Vaccibody immunization

**Figure 2:**
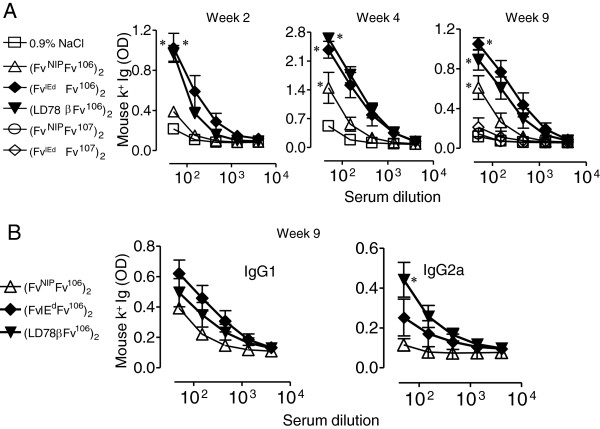
**Levels of anti-Id antibodies elicited by DNA vaccination and electroporation depend upon ability of translated fusion proteins to target APC. (A)** Mice received a single DNA immunization plus electroporation of the indicated plasmid DNA or NaCl control. Sera obtained at different time-points were tested in ELISAs for antibodies binding CLL106 monoclonal IgM/λ purified from heterohybridomas established from patient CLL106 CLL cells. Results at week 2, 4 and 9 after single immunization are shown. Mean ± SEM are plotted, n/group = 7. * p < 0.05 for (F_v_^IEd^F_v_106)_2_*vs* (F_v_^NIP^F_v_106)_2_, (LD78β F_v_106)_2_*vs* (F_v_^NIP^F_v_106)_2_ and (F_v_^NIP^F_v_106)_2_*vs* 0.9% NaCl **(B)** IgG1 and IgG2a components in week 9 sera of Figure 2A. * p < 0.05 for (LD78β F_v_106)_2_*vs* (F_v_^IEd^F_v_106)_2_.

Sera from mice that had been immunized once with different VB constructs were tested for recognition of the corresponding tumor Ig by ELISA at week 2, 4 and 9 after immunization. To this end, the BCR of patient CLL106 was expressed by rescue hybridoma as a secreted IgM used for coating ELISA plates. Improved antibody responses were observed in all mice immunized with either MHC class-II- or chemokine receptor-targeted VB, as compared with mice immunized with the control non targeted NIP-specific VB (Figure 2A and data not shown). By week 9, the effect of targeting was less striking but nevertheless observed, in keeping with previous findings [10,13,16,17,37]. Induction of antibodies was specific for the antigenic unit in the immunogen since sera from mice that had been immunized with VB encoding patient CLL107 scFv Id as antigenic unit did not recognize patient CLL106 IgM in ELISA at any time point (Figure 2A and data not shown). In order to assess the nature of the antibody response induced by the different Vaccibodies, the IgG1 and IgG2a components were compared at different time points. The targeting unit appeared to influence antibody isotype since a trend toward a predominant IgG2a or IgG1 antibodies were observed in sera of mice that had been immunized with chemokine receptor- or MHC class II-targeted VB, respectively, starting from week 4 and peaking at week 9 (Figure 2B and data not shown), in line with previous reports [10,15,37,38].

**Figure 3:**
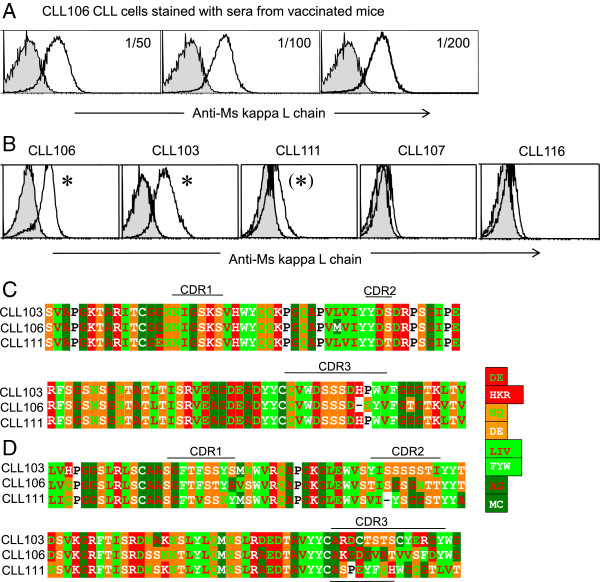
**Antibody responses elicited by targeted Id-DNA vaccines recognize the patient’s CLL cells and cross-react to other patients’ CLL cells expressing highly similar monoclonal Ig. (A)**. Sera from mice that had been DNA vaccinated with the patient CLL106-specific VB targeted by LD78β, were tested in flow cytometry for binding to patient 106 CLL cells. Shaded histogram, serum from mock immunized mouse, black line serum from a representative immunized mouse (n = 5/group). **(B)** The same sera were tested for cross reactivity against a panel of CLL cells obtained from different patients (see Table 1). Data for a representative mouse serum are shown at 1:200 dilution. **(C)** &**(D)** Alignment of V_L_**(C)** and V_H_ regions **(D)** from patients CLL106, CLL103 and CLL111. V regions amino acid sequences were aligned using GeneDoc. Amino acids are color coded according to charge (negative: D, E; positive: H, K, R), or the chemical properties of side chains (i.e. amide: N, Q; alcohol: S, T; aliphatic: L, I, V; aromatic: F, Y, W; small size: A, G; sulfur atom: M, C; or other: P, see key for color code).

We next investigated if sera from the targeted VB 106-immunized mice bound BCR on CLL cells. Sera were admixed with PBMCs from patient CLL106 as well as four other CLL patients (Table 1). Sera from all immunized mice had antibodies binding 106 CLL cells in titres >200 (Figure 3A and data not shown). Strikingly, the sera also cross-reacted with CLL cells from CLL103 and to a lesser extent with CLL111 CLL cells, but not with CLL107 and CLL116 CLL cells (Figure 3B). The CLL cells from these two patients (CLL103, CLL111) expressed Vλ and VH genes of the same family as CLL106 (Table 1 and Figure 3). At the protein level, the L chain V-regions of CLL111 and CLL103 CLL cells were close to identical with CLL106 (Figure 3C). Moreover, the VH were of the same family (IGHV3) and CLL cells were identical to CLL106 in 82% (CLL103) and 83% (CLL111) of the VH amino acids (Figure 3D). The results suggested that the elicited antibodies cross-reacted to homologous sequences or combinatorial V_H_/V_L_ determinants. The lower level of staining of CLL111 cells could be explained by IGLV3-21 being diluted by a second productive L chain rearrangement found in these CLL cells (IGKV3-20*01 F/IGKJ1*01 F) expressed by the vast majority of cells (Table 1, Figure 3B and data not shown), reducing the level of V_L_ and V_H_/V_L_ determinants on these cells.All CLL cells tested expressed low but comparable levels of surface Ig (data not shown). The lack of unspecific staining due to recognition of human IgM by mouse antibodies was ruled out by lack of staining of patient CLL107 and CLL116 cells (Figure 3B and data not shown).

### Construction of a mouse model for B cell lymphomas expressing a prototypic human HCV-associated stereotyped BCR

**Figure 4:**
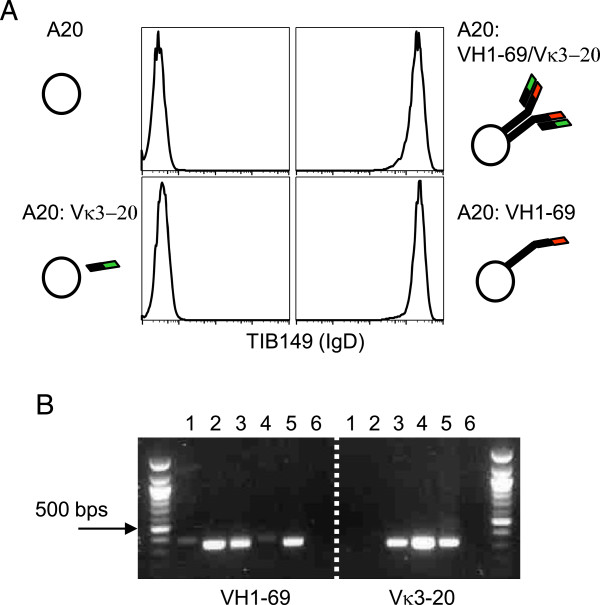
**Construction of mouse B lymphoma cells that express surface chimeric BCR with stereotyped V regions of patients with HCV-associated NHL. (A)** Detection of δ-chains expressing IGHV1-69 on A20 cells transfected with IGHV1-69 and IGKV3-20 (top right), IGVH1-69^only^ (bottom right), IGKV3-20^only^ (bottom left) and untransfected control (top left). IgD staining is shown. Endogenous IgG2aκ is not shown. **(B)** Expression of human IGKV3-20 was verified by RT-PCR. IGHV1-69 and IGKV3-20 amplification: lane 1: parental A20; lane 2: A20 IGHV1-69; lane 3:, A20 IGHV1-69/IGKV3-20, lane 4: A20 IGKV3-20; lane 5:,IGHV1-69 or IGKV3-20 encoding plasmid; lane 6: no template DNA. 100 bp DNA ladder was used.

A sizeable proportion of B cell lymphoproliferative diseases, particularly HCV-associated B cell lymphomas, express IGHV1-69 and IGKV3-20 [18,20]. Thus, we identified prototypic IGHV1-69 and IGKV3-20 genes obtained from two different HCV-associated lymphomas selected as having a representative predicted amino acid sequence. Hence, we constructed a mouse model suitable for the development of an Id vaccine expressing these stereotyped V regions. In order to reduce the possibility that immunocompetent mice spontaneously rejected cells expressing human Ig, prototypic IGHV1-69 and IGKV3-20 V (D) J from patient 1 and 2, respectively, were cloned with mouse δ and κ constant regions, respectively, and transfected into the BALB/c B cell lymphoma A20. It should be noted that the mouse δ construct was engineered so that it was only expressed on the membrane but was not secreted [36]. The following three human/mouse Ig transfectants were generated: (*i*) A20 IGHV1-69, transfected with IGHV1-69 alone, (*ii*) A20 IGKV3-20 transfected with IGKV3-20 alone, and *iii*) A20 IGHV1-69/IGKV3-20 transfected with both IGHV1-69 and IGKV3-20 (Figure 4A). In addition to the transfected chains, A20 cells express an endogenous IgG2a,κ and all types of transfected cells retained expression of the endogenous BCR (data not shown). Surface expression of transfected IGHV1-69-mouse Cδ hybrid gene was confirmed by flow cytometry (Figure 4A). Note also that the expression of IgD/IGHV1-69 in the absence of IGKV3-20 (Figure 4A, bottom right) was detected at the same (or higher) levels than in the presence of VK3-20 (Figure 4A, top right). As A20 cells express endogenous κ chains (κ^Endo^) IgD/ IGHV1-69 could pair with this κ^Endo^.

The lack of a validated anti-human IGKV3-20 mAb negated flow cytometric analysis of V_Κ_ expression, which was therefore screened by RT-PCR (Figure 4B). Thus, no formal proof of surface expression of the human IGHV1-69/IGKV3-20 pair on transfected A20 cells could be obtained, although this is likely to be the case. Transfectants *bona fide* expressing human IGHV1-69/IGKV3-20 were sorted three times to further select efficiently transfected cells.

### Analysis of antibodies induced by DNA immunization with Vaccibodies expressing a stereotyped BCR found in HCV-associated B lymphomas

**Figure 5:**
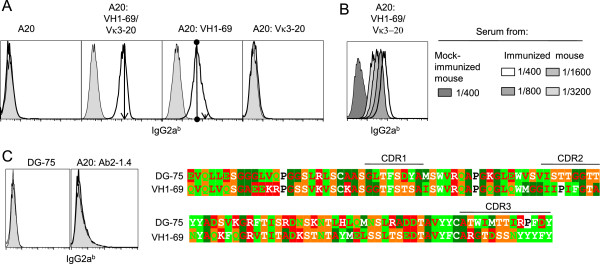
**Chemokine-receptor-targeted Vaccibodies expressing HCV-associated lymphoma prototypic BCR elicit high titer antibodies that bind transfected A20 cells expressing HCV-associated lymphoma prototypic BCR.** Five Ig haplotype IgH-C^b^ mice (B10.D2-TCRα^a^) were DNA vaccinated with LD78β VB having as antigenic unit prototypic HCV-associated B lymphoma BCR (with IGHV1-69 and IGKV3-20 linked in a scFv format), followed by electroporation. **(A)** Sera containing IgG^b^ obtained 21 days later were used at 1:400 dilution to stain both transfected and parental A20 cells (that express Ig haplotype IgH-C^a^), bound IgG2a^b^ was detected with an mouse anti-IgG2a^b^ mAb. Representative staining are shown of immunized sera (line) or control (gray filled). Peak channels are indicated in the second (arrows) and third panels (line-circles). For comparison, an arrow in the third panel indicates the peak channel from the second panel. **(B)** Antibody titres of a representative mouse. **(C)** Left: sera from a mock immunized (gray filled) or a representative immunized mouse (line) did not stain cells from the IGKV3-20^+^ Burkitt’s lymphoma (DG-75), or A20 expressing syngeneic V regions on BALB/c IgD,κ scaffold (Ab2-1.4). Right: Comparison of the VH amino acid sequence of DG-75 and IGHV1-69. Amino acids are indicated as in Figure 3.

Mice were immunized with LD78β-VB having an antigenic unit comprised of prototypic HCV-associated B lymphoma BCR with IGHV1-69 and IGKV3-20 linked in a scFv format. In terms of secondary detection of bound murine antibodies, it is notable that A20 cells endogenously express surface IgG2a, precluding detection of bound serum IgG2a. Immunized mice had low levels of serum IgG1 antibodies that stained IGHV1-69/IGKV3-20 A20 cells (data not shown). To be able to visualize IgG2a responses, we also immunized a strain of mice that differ in Ig H-chain allotype, permitting use of anti-IgG2a allotype-specific antibodies in flow cytometry. Hence, B10.D2.C-TCRα^a^ (H-2^d^, Ig haplotype IgH-C^b^) mice (see Materials) were immunized with the same VB as above. Sera from mice that had been immunized once bound A20 IGHV1-69/IGKV3-20 as well as A20 IGHV1-69 cells, but not untransfected A20 or A20 IGKV3-20 cells (Figure 5A). Antibody titres were more than 1:3200 (Figure 5B). Note that as A20 expresses endogenous gamma chains (γ^Endo^) as well as κ (κ^Endo^), the latter cells could express γ^Endo^-chains paired with IGKV3-20. Similarly, the IGHV1-69 transfectant could express this heavy chain paired with κ^Endo^. We further tested sera for binding to DG-75 [31], a sIgMκ^+^ Burkitt’s lymphoma cell line that expresses very similar IGKV3-20 (93.8% identity of the Vκ amino acids) but dissimilar VH (IGHV3-23) (only 51.5% identity of VH amino acids) (Figure 5C and data not shown). In spite of the expression of the correct IGKV3-20 L chain, sera failed to stain these cells, suggesting that the elicited antibodies were predominantly specific for V_H_. However, some anti-Id antibodies most likely bound V_L_/V_H_ combinatorial determinants since A20 cells expressing VH1-69/VK3-20 stained brighter than A20 cells expressing only VH1-69. This result was obtained with sera from 4 out of 5 mice (Figure 5A, arrows/circles), indicating a contribution of combinatorial V_H_/V_L_ epitopes. The sera from all mice failed to bind A20 cells expressing an unrelated IgD (Ab2-1.4, see Methods and Figure 5C), as well as a Burkitt’s lymphoma control (PA682, see Methods).

### Growth of transfected A20 cells in BALB/c mice

Three BALB/c mice per group were injected s.c. with 3 × 10^6^ either parental A20 cells or IGHV1-69/IGKV3-20 transfected A20 cells. Two out of three mice that had been injected with parental A20 cells developed tumors, whereas no mice that had been injected with IGHV1-69/IGKV3-20 A20 cells developed tumors, presumably due to immunogenicity of the xenogeneic (human) Ig V regions sequences in mice. These results are reminiscent of our previous finding in multiple myeloma, where the tumorigenicity of MOPC315.36 was decreased by stable expression of human V genes [17]. The lack of tumorigenicity of the A20 transfectant in immunocompetent mice precluded both prophylactic and therapeutic antitumor vaccination experiments.

## Discussion

Herein we describe experiments aimed at generating Id vaccines for therapeutic Id vaccination of groups of patients with B cell malignancies expressing stereotyped BCRs. First, we demonstrate the feasibility of constructing fully human Id Vaccibodies with maintenance of both Id epitopes and functionality of targeting units (i.e., human CCL3 chemokine LD78β that cross-react with mouse CCRs) [16]. Similar to fully murine and chimeric mouse/human vaccibodies, targeting antigen delivery to APC by human chemokine resulted in augmented immune responses in mice as compared with non-targeted control DNA Vaccibodies [16]. Since these vaccines are fully human and isoform LD78β of human CCL3 bind cells expressing *Rhesus macaque* CCR5 [16], they are suited for both preclinical immunogenicity and regulatory toxicology studies in view of clinical application. It may be anticipated that targeting scFv Id to APC by LD78β could result in increased anti-Id responses in patients. Furthermore, DNA vaccination combined with electroporation is already employed in clinical trials for melanoma and prostate cancer, with mild to moderate, reversible side effects [39].

Second, we explore a complementary approach to further streamline clinical application of Id vaccine for B cell malignancies.

Antibodies elicited in mice by Id DNA Vaccibodies constructed for a CLL patient showed cross-reactivity with CLL cells from some other patients. The most plausible explanation is that a fraction of mouse antibodies recognized epitope(s) displayed by the V_L_ and/or V_L_/V_H_ of the cross-reactive CLLs, since the CLL cells expressed nearly identical IGLV3-21 and similar IGHV3 family genes. With the HCV-NHL construct, the induced antibodies bound the transfected A20 cells but failed to bind a Burkitt’s lymphoma expressing the correct Vκ but not the corresponding VH, indicating a dominant anti-VH response. Even so, a contribution of the Vκ was however detected as VK3-20^+^VH1-69^+^ transfectants stained brighter than Vk^Endogenous^/VH1-69^only^ cells (that express an endogenous Vκ), suggesting responses towards VL/VH combinatorial idiotypes. Taken together, it is suggested that immunization of mice with fully human targeted scFv Id DNA vaccines could elicit antibodies that may react with either V_L_, or V_H_, or V_L_ + V_H_, the relative proportions differing from case to case.

The above observations suggest the possibility of constructing vaccines covering molecularly identified subgroups of patients with B cell malignancies. This idea is supported by evidence of Id cross-recognition by anti-Id mAb [40] as well as cross-reactive responses observed in clinical trials [41-45]. A subgroup-specific, “off-the-shelf” Id vaccine should elicit a cross-reactive immune response effective against unrelated B cell tumors expressing V regions of the same families, provided that the pattern of somatic mutations is similar between individual tumors. In this respect, the application of criteria developed for clustering stereotyped BCR as based on HCDR3 sequences only [27] do not fully meet the need for identifying patients amenable to immunization with such vaccines, as immune responses following vaccination may be directed to determinants located elsewhere in the V regions [45-47]. Hence, similarity across the whole V regions should be evaluated. In principle, staining of lymphoma sections or single cell suspensions obtained from biopsy with serum from mouse that had been immunized with the intended Id vaccine could be able to identify candidate patients.

Evidence of clinical benefit by Id vaccination has been obtained so far only upon immunization with whole Ig protein vaccine [2,3], thus displaying to the host immune system both tumor-specific V_H_ and V_L_. Therefore, considering that IGHV1-69 is often the partner of IGKV3-20 in HCV-related NHLs [18,20], a prototypic BCR for a subset of HCV-associated NHL was cloned, inserted into VB format and used to DNA immunize mice. We chose IGHV and IGKV from unrelated lymphomas purposely considering the possible use of such Vaccibodies as off-the-shelf, subgroup-specific vaccines. In fact, while HCV-associated NHL express IGHV1-69 and IGKV3-20 proteins with high similarity in the framework regions, the presence of several differences in the amino acid sequence of the CDR regions makes it difficult to select a one-for-all IGHV-IGKV pair. On these grounds, we selected IGHV1-69 and IGKV3-20 proteins as prototypic on the basis of their representativity of the FR regions among HCV-associated lymphomas. Our results show that such an artificial targeted DNA Id vaccine elicits antibodies in mice that bind mouse B lymphoma cells transfected with the H and L chain genes composing the artificial BCR. Whether these antibodies bind human IGHV1-69/IGKV3-20 B lymphomas remains to be investigated pending sample availability.

From a translational standpoint, the possibility of using DNA vaccines encoding scFv with the potential to elicit cross-reactive immune responses is not restricted to the IGHV1-69/IGKV3-20 combination, as similar features of conserved V regions usage have been detected in other B cell malignancies. Thus, one can envision tailored Id vaccines for each major stereotyped subset identified. To estimate the number of patients with B-cell malignancies that could be immunized with off-the-shelf cross reactive vaccines, a large database including sequences of idiotypic VH and VL genes expressed by low grade B-NHL, autoimmunity-associated lymphoproliferations (e.g. HCV-related NHL, mixed cryoglobulinemia, Sjögren’s syndrome) and CLL is currently being set up with the aim of identifying subgroups of tumors characterized by the expression of molecularly correlated Id proteins on the basis of the degree of sequence conservation among patients (R Dolcetti, unpublished results). However, stereotyped BCR sequences appear to be disease-biased. In CLL, shared V regions are in most cases unmutated [25,26,48,49] whereas in other B cell tumors (e.g., HCV-associated lymphomas, MALT lymphomas) somatic mutations are more frequent [18,20,21]. Therefore, from an immunological standpoint, the yet unanswered question as to whether the host immune system can recognize V region sequences in germline configuration following Id vaccination is of paramount relevance for the possibility of applying Id vaccines, whether individual or subgroup-specific [50].

It should be stressed that certain human B cell malignancies have not been described to express stereotyped BCR. One example is multiple myeloma cells that carry high loads of somatic mutations in their V regions, consistent with an origin from post germinal center B cells [51]. In a previous report, we demonstrated that mice DNA-immunized with hybrid mouse/human Vaccibodies expressing scFv of either of four myeloma patients induced anti-Id antibodies that bound the corresponding myeloma protein with little cross-reactivity despite the fact that the BCR of two patients used the same IGHV and IGHJ genes [16]. Thus, in the case of multiple myeloma, V regions of monoclonal Ig express unique Ids with little cross-reactivity, at least as defined by antibodies elicited by APC-targeted DNA Id vaccines.

The discussion above has focused on antibodies elicited by APC-targeted DNA Id vaccines since anti-Id antibodies have been linked to anti-lymphoma activity both in passive and active immunotherapy [52,53]. However, MHC-restricted, Id-specific T cells have been shown to display anti-lymphoma activity and to eradicate B cell tumors [43,47,54,55]. scFv in the vaccine contains Id-sequences available for MHC presentation. T cell responses were not investigated in this study as they would have been directed against xenogeneic Ig sequences thus having no semblance to the clinical situation. Nevertheless, it can be speculated that subgroup-specific vaccines can elicit cross-reactive Id-specific T cell responses which may or may not be accompanied by cross-reactive humoral responses, as has recently been suggested with HCV-associated B cell lymphoma IGKV3-20 [56], However, patients differ in polymorphic HLA molecules and are therefore expected to present different sequences of V regions of CLL/B lymphoma BCR on their HLA molecules, thus making the possibility of cross-reactive T cell responses less common as compared with antibody responses. Also, based on mouse studies, T cell tolerance to the CLL/B lymphoma BCR is likely to limit T cell responsiveness in humans to a greater extent than humoral responses.

## Conclusions

Herein we demonstrate the feasibility of constructing fully human Vaccibodies that target scFv Id to mouse APC *in vivo* enabling enhanced immune responses. The striking similarity of amino acid Id sequence found across different subgroups of patients affected by molecularly identified B cell malignancies could be exploited to prepare subgroup-specific vaccines. As opposed to patient-specific vaccines, such “off-the-shelf” vaccines could reduce the number of tailor-made Id DNA vaccines allowing substantial time and cost savings.

## Abbreviations

Ab: Antibody; Ag: Antigen; APC: Antigen-presenting cells; BCR: B Cell receptor; CLL: Chronic lymphocytic leukemia; Fab: Fragment antigen binding; Id: Idiotype; Ig: Immunoglobulin; HCV: Hepatitis C virus; HLA: Human Leukocyte Antigen; MHC: Major histocompatibility complex; NHL: Non Hodgkin’s lymphoma; scFv: Single chain fragment variable; V: Variable; VB: Vaccibody.

## Competing interests

BB and PAR are inventors of Vaccibody patent applications filed by their employer (Univeristy of Oslo and Oslo University Hospital). BB is head of the scientific panel of the company Vaccibody AS.

## Authors’ contributions

PAR, BB and RD conceived of the study. PAR carried out the construction and *in vitro* characterization of the vaccines, performed the mouse immunization studies, and drafted the manuscript. AO and LAM contributed to the CLL analysis and experiments. GET provided CLL patient samples, V region sequencing and clinical information. RD provided the plasmids for HCV-associated NHL BCR Vaccibody and helped to draft the manuscript. BB participated in the study design and coordination and helped to draft the manuscript. PAR, LAM and BB wrote the final manuscript. All authors read and approved the final manuscript.
